# Detection of Novel Rotavirus Strain by Vaccine Postlicensure Surveillance

**DOI:** 10.3201/eid1908.130470

**Published:** 2013-08

**Authors:** Geoffrey A. Weinberg, Elizabeth N. Teel, Slavica Mijatovic-Rustempasic, Daniel C. Payne, Sunando Roy, Kimberly Foytich, Umesh D. Parashar, Jon R. Gentsch, Michael D. Bowen

**Affiliations:** University of Rochester School of Medicine and Dentistry, Rochester, New York, USA (G.A. Weinberg);; Centers for Disease Control and Prevention, Atlanta, Georgia, USA (E.N. Teel, S. Mijatovic-Rustempasic, D.C. Payne, S. Roy, K. Foytich, U.D. Parashar, J.R. Gentsch, M.D. Bowen)

**Keywords:** rotavirus, viruses, gastroenteritis, phylogeny, child, enteric infections, G14P[24], strain, diarrhea, vaccines, surveillance

## Abstract

Surveillance for rotavirus-associated diarrhea after implementation of rotavirus vaccination can assess vaccine effectiveness and identify disease-associated genotypes. During active vaccine postlicensure surveillance in the United States, we found a novel rotavirus genotype, G14P[24], in a stool sample from a child who had diarrhea. Unusual rotavirus strains may become more prevalent after vaccine implementation.

Active vaccine postlicensure surveillance for rotavirus-associated diarrhea is informative for determination of vaccine effectiveness and for characterization of disease-associated rotavirus genotypes (*1*–*5*). Most rotaviruses circulating in the United States belong to a limited number of strains, routinely characterized by serologic or genetic identification of the outer capsid protein antigens viral protein (VP) 7, which defines G types, and VP4, which defines P types (*6*,*7*). Of circulating strains in the United States, 85% contain a G or P antigen that is included in both US-licensed rotavirus vaccines (*2*,*8*). However, >70 G and P antigen combinations have been reported, and uncommon strains may suddenly appear in a new geographic area (*1*,*2*,*5*,*9*). Ongoing active surveillance is conducted through the Centers for Disease Control and Prevention’s New Vaccine Surveillance Network, a prospective, population-based surveillance program for acute gastroenteritis among children <5 years old, the details of which have been published (*3*–*5*). This surveillance has detected the emergence of G12P[8] and G9P[8] rotavirus genotypes, as well as 3 reported instances of US children infected with G8P[4] rotavirus (*3*–*5**,**10*). During the 2009 winter season (December 2008–June 2009) in Rochester, New York, 54 (30%) of 183 enrolled children with acute gastroenteritis had rotavirus infection. Fifty (94%) of 51 rotavirus strains were typical US strains, with G or P antigens contained in the licensed rotavirus vaccines; 3 were G8P[4] (*10*). One strain, however, appeared to be an unusual reassortant not previously reported in human infection. We describe this novel rotavirus genotype, G14P[24], found along with enteric adenovirus in a stool sample from a child with diarrhea.

## The Study

A 36-month-old girl was brought to the emergency department of the Golisano Children’s Hospital at University of Rochester Medical Center with a 4-day history of emesis (2 times/day) and low-grade fever (37.7°C). During the previous 2 days, she also had experienced diarrhea (8 loose stools/day) and lethargy. Physical examination was only remarkable for mild dehydration; there were no other abnormalities. She was previously healthy, born after a full-term gestation, and breast-fed for the first 7–12 months of life. The child had not been vaccinated against rotavirus. She lived in the Rochester metropolitan area and had no unusual dietary or travel exposures. She had contact with pet dogs and cats at home and at a childcare setting. Approximately 1 month before her illness, she had visited a petting zoo at which farm animals but no nonhuman primates were present; her mother could not remember whether horses or cows were present but recalled the child petting sheep. 

After oral rehydration, the child’s activity increased, and she was discharged to home. She continued to experience intermittent emesis and diarrhea for 1 month, although she maintained her weight. No other family members (1 sibling, 2 parents) became ill.

**Figure 1:**
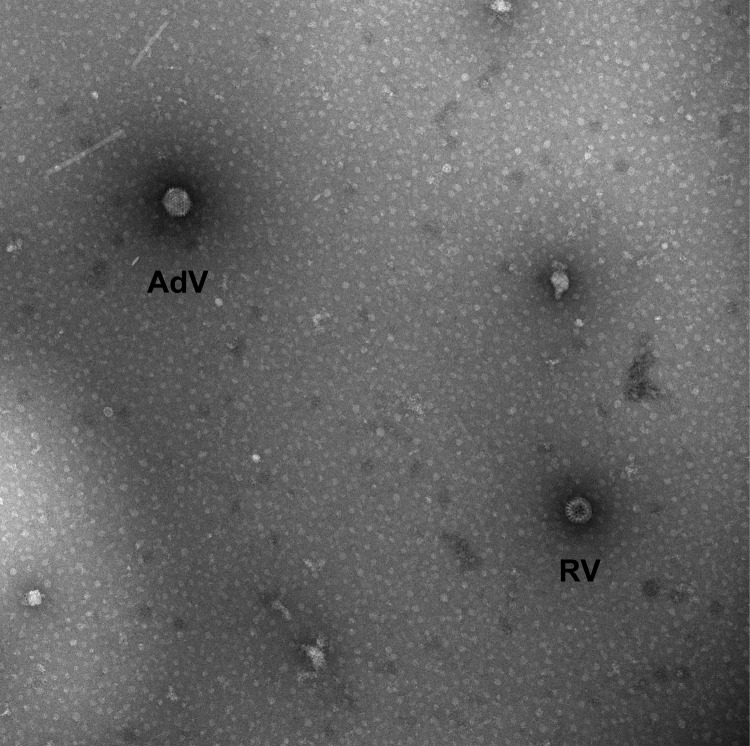
Transmission electron micrograph image of stool sample from 36-month-old child with diarrhea, showing viral particles characteristic of rotavirus (RV) and enteric adenovirus (AdV). Magnification ×92,300. Image courtesy of Charles Humphrey.

The child was enrolled, with parental informed consent, into the New Vaccine Surveillance Network. A stool sample taken during the hospital visit was positive for rotavirus antigen by enzyme immunoassay (Premier Rotaclone; Meridian Bioscience, Inc., Cincinnati, OH, USA). The specimen was analyzed at the Centers for Disease Control and Prevention by transmission electron microscopy, reverse transcription PCR genotyping, and nucleotide sequencing, as described (*11*). Electron microscopy showed 2 types of virions, 1 characteristic of rotavirus and 1 of enteric adenovirus (Figure 1).

**Figure 2:**
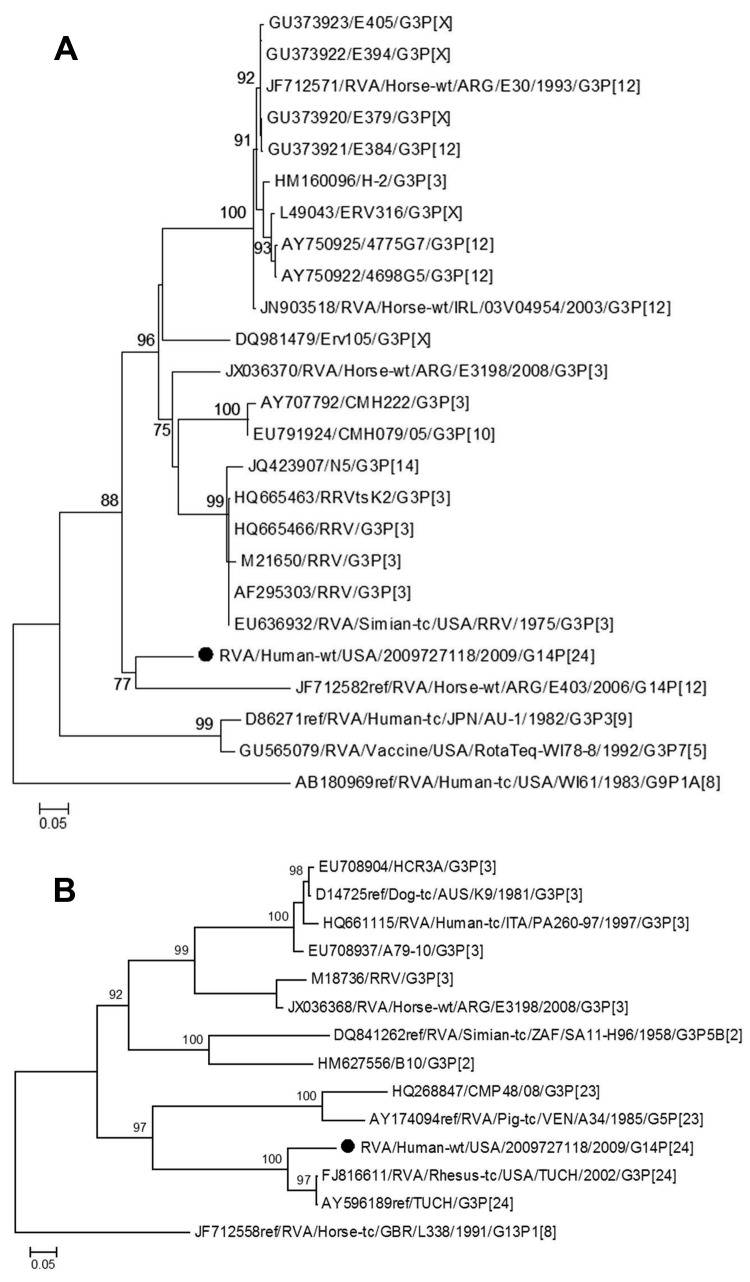
Genetic relationships of partial viral protein 7 (A) and viral protein 4 (B) nucleotide sequences for novel rotavirus strain (black dot) isolated from 36-month-old child with diarrhea compared with representatives of known equine, simian, and human rotavirus genotypes. Evolutionary relationships and distances were inferred by using the maximum-likelihood method in PhyML 3.0 (*13*). Numbers next to nodes are approximate likelihood-ratio test values calculated by PhyML. Rotavirus strain designations, and G and P genotypes are shown. Scale bars indicate number of nucleotide substitutions per site.

Analyses of VP7 and VP4 sequences using RotaC 2.0 (*12*) identified the rotavirus strain as genotype G14P[24] (*6*,*7*) (Figure 2). Phylogenetic analyses indicated monophyly of the VP7 gene with an equine rotavirus strain from Argentina and clustering of the VP4 gene with the simian rotavirus strain TUCH (Figure 2). The novel strain was designated as RVA/Human-wt/USA/2009727118/2009/G14P[24], in accordance with guidelines from the Rotavirus Classification Working Group (*6*,*7*). Full genome sequencing is in progress; the preliminary 11-gene genotype is G14-P[24]-I9-R2-C3-M3-A9-N3-T3-E3-H6, which indicates that this novel strain may be a reassortant containing genes from equine, simian, human, and bovine rotaviruses. In particular, the VP7 gene seems to be most related to equine G14 strains; the VP4 gene, to simian P[24]; the VP1 gene, to bovine R2; and the nonstructural protein 3 gene, to human T3 strains.

## Conclusions

Human rotavirus infection is commonly associated with ≈6 of the >70 known human G and P antigen combinations reported among >160 known rotavirus strains (*1*,*6*). The G14P[24] strain we found had not been reported in human infection, but interspecies transmission of both reassorted and nonreassorted animal viruses has been described (*9*). The emergence of unusual reassortant animal strains raises questions about the effectiveness of current rotavirus vaccines, which might share neither G nor P types with such viruses. However, immunity to rotavirus is believed to be polygenic and probably involves antigens in addition to G and P antigens (*14*).

In summary, we identified infection with a novel G14P[24] rotavirus strain in a 36-month-old child with diarrhea. Whether this strain was responsible, entirely or in part, for the child’s symptoms is not certain, because enteric adenovirus was also identified. Co-infection with rotavirus and enteric adenovirus has been described, but it is unclear whether such co-infection is associated with more severe gastroenteritis (*15*). Nevertheless, the rotavirus strain we identified appears to be an unusual reassortant containing equine, human, simian, and bovine rotavirus genes. Further study of this and other unusual reassortant rotaviruses may lead to insight on rotavirus evolution. Continued surveillance is critical for assessing whether unusual genotypes of rotavirus become more prevalent after the implementation of rotavirus vaccination.
